# Association between Life’s Simple 7 and cerebrospinal fluid biomarkers of Alzheimer’s disease pathology in cognitively intact adults: the CABLE study

**DOI:** 10.1186/s13195-022-01019-2

**Published:** 2022-05-26

**Authors:** Yong-Li Zhao, Ya-Nan Ou, Ya-Hui Ma, Yu-Yuan Huang, Yan-Lin Bi, Lan Tan, Jin-Tai Yu

**Affiliations:** 1 Department of Neurology, Qingdao Municipal Hospital, Qingdao University, No. 5 Donghai Middle Road, Qingdao, China; 2 Department of Neurology and Institute of Neurology, State Key Laboratory of Medical Neurobiology and MOE Frontier Center for Brain Science, Huashan Hospital, Shanghai Medical College, Fudan University, Shanghai, 200040 China; 3 Department of Anesthesiology, Qingdao Municipal Hospital, Qingdao University, Qingdao, China

**Keywords:** Life’s Simple 7, Cerebrospinal fluid, Biomarkers, Alzheimer’s disease, Pathogenesis

## Abstract

**Introduction:**

This study sought to explore the association between Life’s Simple 7 (LS7) and cerebrospinal fluid (CSF) Alzheimer’s disease (AD) pathological biomarkers in the cognitively normal northern Chinese population.

**Methods:**

From the Chinese Alzheimer’s Biomarker and LifestylE (CABLE) study, 1106 cognitively normal participants were enrolled. The mean age was 62.34 years, and 39.6% were female. LS7 scores were summed with each metric assigned 0, 1, or 2 scores. The multiple linear regression models were used to investigate the association between LS7 scores and CSF AD biomarkers.

**Results:**

We found that LS7 scores were significantly associated with CSF AD pathologies, including Aβ42/40 (*β* = 0.034, *P* = .041), p-tau181 (*β* =  − 0.043, *P* = .006), and t-tau (*β* =  − 0.044, *P* = .003). In subscales, the biological metrics (blood pressure, cholesterol, glucose) were significantly related to CSF tau-related biomarkers. These associations were observed in the *APOE* ε4 allele non-carriers, yet not in carriers. The relationship of behavior metrics was found in the middle age and males.

**Conclusion:**

Improving LS7 scores might do a favor to alleviate the pathology of AD in the preclinical stage, especially among the *APOE* ε4 allele non-carriers.

**Supplementary Information:**

The online version contains supplementary material available at 10.1186/s13195-022-01019-2.

## Introduction

As the most common form of dementia, Alzheimer’s disease (AD) is a multifactorial neurodegenerative disease, featured by extracellular amyloid β plaques (Aβ) and intracellular tau neurofibrillary tangles of the brain which could have changed decades before the clinical stage and can be detected by biomarkers of positron emission tomography (PET) imaging and cerebrospinal fluid (CSF) [1, 2]. Since there is no effective treatment, prevention remains the most preferred and earliest strategy. Most studies have focused on a single factor of individual lifestyle and vascular risks, which have been identified as modifiable factors and could only approximately attribute a third of dementia cases [3]. However, recent evidence suggested that multidomain intervention simultaneously might play more roles in preventing cognitive decline and dementia [4, 5].

The Life’s Simple 7 (LS7) was proposed as an assessment of cardiovascular health by the American Heart Association (AHA), based on 4 health behaviors (physical activity, body mass index [BMI], diet, and smoking) and 3 biological metrics (blood pressure [BP], fasting blood glucose [FBG], and total cholesterol [TC]) [6]. Emerging proofs suggested that ideal LS7 was associated with better cognition [7–9]. Moreover, it was related to reduced risks of dementia and AD [5, 10–12]. Nonetheless, insignificant relationships between LS7 and cognition, dementia, and AD were also observed [13–16]. The correlations between LS7 and white matter hyperintensity, silent brain infarct, cerebral volume, and higher total brain and gray matter volumes have been observed [10, 17]. However, the relationships between LS7 and AD core pathologies have not been examined, including Aβ42, total tau (t-tau), and phosphorylated tau (p-tau) which could be well reflected in CSF with decreased Aβ42 and increased t-tau and p-tau181 levels [18]. Nonetheless, the heterogeneity of CSF Aβ42 levels was thought significant [19], and Aβ42/40 ratio was believed to improve the accuracy of discriminating AD compared to Aβ42 [20]. CSF Aβ40 is the most bountiful variation of Aβ, yet it is less pathological. Many studies showed that CSF Aβ40 levels were higher in populations with AD, indicating that it may serve as a clue to AD risks [21, 22]. Therefore, in this study, we aimed (1) to research the associations between LS7 and AD core hallmarks of CSF Aβ42, Aβ42/40, t-tau, and p-tau181 and the biomarker of CSF Aβ40 in cognitively intact adults and (2) to research the above relationship among different *APOE* ε4 allele statuses, age groups, and genders.

## Materials and methods

### The populations

All individuals in this study were enrolled in the Chinese Alzheimer’s Biomarker and LifestylE (CABLE) study, a large-scale ongoing study from 2017 exploring the genetic and environmental factors and biomarkers of AD in the 40- to 90-year-old northern Han Chinese population [23]. The subjects were recruited from the city of Qingdao, Shandong province, China, with a convenient sample from hospitalized patients of Qingdao Municipal Hospital. The exclusion criteria were (a) infection of the central nervous system, epilepsy, head trauma, major neurological disorders, or other neurodegenerative diseases rather than AD (e.g., Parkinson’s disease); (b) major psychological disorders; (c) severe systemic diseases (e.g., malignant tumors); and (d) family history of genetic diseases. The Institutional Ethics Committee of Qingdao Municipal Hospital approved the CABLE study, and it was carried out following the Declaration of Helsinki. All subjects or their proxies gave written consent.

A total of 1106 cognitively intact subjects with adequate data of CSF biomarkers and LS7 measurements were enrolled in this cross-sectional study. All individuals underwent comprehensive clinical, psychiatric neuropsychological examinations; biochemical testing; and biological samples (blood and CSF sample) collections at study entry. The basic information of age, sex, years of education, and medical history was obtained through a structured questionnaire and supplemented by an electronic medical record system. Global cognitive function was examined by the China Modified Mini-Mental State Examination (CM-MMSE). Depression and anxiety were assessed using the Hamilton Rating Scale for Depression (HAMD) and Hamilton Rating Scale for Anxiety (HAMA), respectively. The population diagnosed with cognitive impairment (CM-MMSE ≤ 24 for > 6 years of education, ≤ 20 for no more than 6 years of education, ≤ 17 for no education), significantly depression (HAMD > 7), or anxiety (HAMA > 7) were excluded.

### CSF AD biomarkers assessments and APOE-ε4 genotyping

The fasting CSF sample was extracted via a standard operating procedure with the discarding of the first 1–2 mL and processed within 2 h. After centrifuging at 2000 × *g* for 10 min, it was stored in an enzyme-free EP (Eppendorf) tube at − 80 °C. The thaw/freezing cycle was limited to two times or less. CSF Aβ42, Aβ40, p-tau181, and t-tau levels were determined with the ELISA kits (Innotest β-AMYLOID (1–42) [catalog number: 81583]; β-AMYLOID (1–40) [catalog number: 81585]; PHOSPHO-TAU (181p) [catalog number: 81581]; hTAU-Ag [catalog number: 81579]; Fujirebio, Ghent, Belgium). All measurements were performed by professional experimenters who were blind to clinical information. The within-batch coefficient of variation (CV) was < 5% (mean CV 4.5% for Aβ42, 3.7% for Aβ40, 2.5% for p-tau181, and 4.4% for t-tau). The inter-batch CV was < 20% (mean CV 5.3% for Aβ42, 3.4% for Aβ40, 2.4% for p-tau181, and 4.8% for t-tau).

Using the QIAamp® DNA Blood Mini Kit (250), DNA was drawn from fasting blood samples. Next, it was separated and stored in an enzyme-free EP tube at − 80 °C until the *APOE* genotyping was completed in this study. Two specific loci related to *APOE* status (rs7412 and rs429358) were selected for genotyping with restriction fragment length polymorphism technology. Participants were classified as *APOE* ε4 non-carriers and *APOE* ε4 carriers (individuals with at least one copy of the *APOE* ε4 gene).

### Measurements of LS7 metrics

We looked into each indicator of LS7 and categorized them into poor (score as 0), intermediate (score as 1), and ideal (score as 2) qualities leaning on the AHA criteria and with modifications in terms of diet and physical activity (Additional file 1: Table S1). All of the behavior metrics including BMI, smoking, diet, and physical activity were measured through a self-reported questionnaire and medical record system. The biological metrics of BP, cholesterol, and glucose levels were tested by professionals in the laboratory.

We obtained BMI by the calculation of weight divided by height squared. Subjects who were smoking were regarded as current smokers, and those who had quit smoking were regarded as past smokers with different quitting times. Compared with the AHA criteria, our diet metric only involved two components of fruit and fish, and with the information of frequency through a questionnaire, yet lacking quantitative data. Moreover, the whole grain, sodium, and sugar-sweetened beverage intake were not included since insufficient information was collected. For each ingredient, the daily frequency was coded as 2, once or several times a week coded as 1, and never or occasionally coded as 0, and the sum of the two ingredients was used to ultimately categorize the diet metric (Additional file 1: Table S1). The measurement of physical activity during leisure time was examined through a questionnaire looking at the frequency of exercise, with a major limitation of lacking intensity and duration. We assigned daily frequent as 2 scores, once a week or several times a week as 1 score, and never or occasionally as 0 scores. BP was measured in triplicate every morning when the participants were resting and in a sitting position during their first 5 days of hospitalizations, and the mean of measurements was used to divide the metric. After fasting for at least 8 h, the enzymatic method and the glucose hexokinase (HK) method were used to test the fasting plasma total cholesterol and glucose levels, respectively.

The composite LS7 scores were calculated from the sum of 7 components, ranging from 0 to 14. It was further ranked as poor (scores 0–5, < mean − standard deviation [SD]), intermediate (scores 6–10, ≥ mean − SD and < mean + SD), and optimal (scores 11–14, ≥ mean + SD) levels.

### Statistical analysis

The baseline characteristics were compared using the chi-square test (for categorical variables) and the analysis of variance or Kruskal–Wallis test (for continuous variables). We normalized the level of CSF AD biomarkers by the Box–Cox transformations using the “car” package of the R software and standardized them by *Z*-scale in case of skewed distribution. Extreme values outside the 3 SD of CSF AD biomarkers were excluded.

Across the three categories of LS7 scores, differences in CSF AD biomarkers were compared using analysis of variance, and the cognition test was compared using the Kruskal–Wallis test. We applied multiple linear regression (MLR) models to explore the association between total LS7 scores and CSF Aβ42, Aβ42/40, Aβ40, t-tau, and p-tau181 biomarkers, with the adjustment of age, sex, education, and *APOE* ɛ4 allele statuses. Moreover, the subscale of the biological metrics (the summary scores of BP, total cholesterol, and glucose), behavior metrics (the summary scores of BMI, smoke, diet, and physical activity), and individual components of LS7 was investigated. Interactions were tested in regression models by the terms of *APOE* genotype, age, and sex with LS7 score, and followed by stratified analyses by different *APOE* ε4 allele statuses (non-carrier or carrier), mid-life (< 65 years), or late life (≥ 65 years), male or female. Lastly, sensitivity analyses were performed by (1) additionally adjusting for the comorbidities of coronary heart disease and stroke to control relevant confounders and (2) analyzing the relatively healthy population with no history of hypertension, diabetes, and hyperlipemia, who were in an early stage of disease, to validate the association between LS7 score and CSF AD biomarkers.

The packages “car,” “ggplot2,” and “lm” in the R 4.0.3 software were used for statistical analyses and illustrations (R Project for Statistical Computing; http://www.r-project.org). *P* values of less than 0.05 were considered significant.

## Results

### Characteristics of participants

We totally included 1106 subjects with a mean age of 62.34 (SD = 10.27), ranging from 40 to 89, which were shown in Table 1. About one-third (39.6%) of the participants were female, and 137 (13.92%) were *APOE* ɛ4 carriers. A total of 639 were in their middle age (< 65 years), and 467 were in late age (≥ 65 years). The mean LS7 score was 7.99 (SD = 2.05), and the distribution was exhibited in Additional file 1: Fig. S1. Individuals with higher levels of LS7 scores were younger and better educated (Table 1).

**Table 1:** Characteristics of participants across LS7 categories

Characteristics	LS7 categories			Total	*P* value
	**Poor**	**Intermediate**	**Optimal**		
*N*	128	852	126	1106	–
Age (years), mean (SD)	62.12 (9.74)	62.89 (10.15)	58.84 (10.96)	62.34 (10.27)	**.0002**^a^
Gender, female (%)	44 (34.37)	334 (39.20)	60 (47.61)	438 (39.60)	.0861^b^
Education (years), mean (SD)	9.35 (4.33)	9.61 (4.21)	10.90 (3.83)	9.73 (4.20)	**.0031**^a^
*APOE* ε4, yes (%)	19 (16.10)	104 (13.79)	14 (12.50)	137 (13.92)	.7163^b^
CM-MMSE, mean (SD)	27.85 (2.26)	27.90 (2.06)	28.33 (1.77)	27.94 (2.05)	.0942^c^
LS7 score, mean (SD)	4.47 (0.78)	8.01 (1.28)	11.44 (0.70)	7.99 (2.05)	** < .0001**^a^
**CSF AD biomarkers**					
Abeta42, mean (SD)	187.19 (97.19)	202.61 (112.61)	182.53 (91.32)	198.64 (108.95)	.1920^a^
AbetA40, mean (SD)	6116.46 (2834.65)	5880.47 (2650.43)	5014.28 (2349.72)	5808.7 (2653.89)	**.0015**^a^
Abeta42/40, mean (SD)	0.0401 (0.0423)	0.0404 (0.0289)	0.0411 (0.0213)	0.0404 (0.0300)	.2090^a^
P-TAU181, mean (SD)	38.46 (10.45)	37.68 (9.30)	34.25 (6.95)	37.36 (9.26)	**.0005**^a^
T-TAU, mean (SD)	183.77 (78.51)	177.72 (83.35)	149.44 (65.18)	175.21 (81.43)	**.0001**^a^

The statistically significant results have been bolded

*LS7* Life’s Simple 7, *APOE ε4* apolipoprotein E genotype ε4, *CM-MMSE* China Modified Mini-Mental State Examination, *P-TAU181* phosphorylated tau181, *T-TAU* total tau

^a^The difference among the groups was examined by the analysis of variance

^b^The difference among the groups was examined by the chi-square test

^c^The difference among the groups was examined by the Kruskal–Wallis test

### Association between LS7 scores and CSF AD biomarkers

**Fig. 1:**
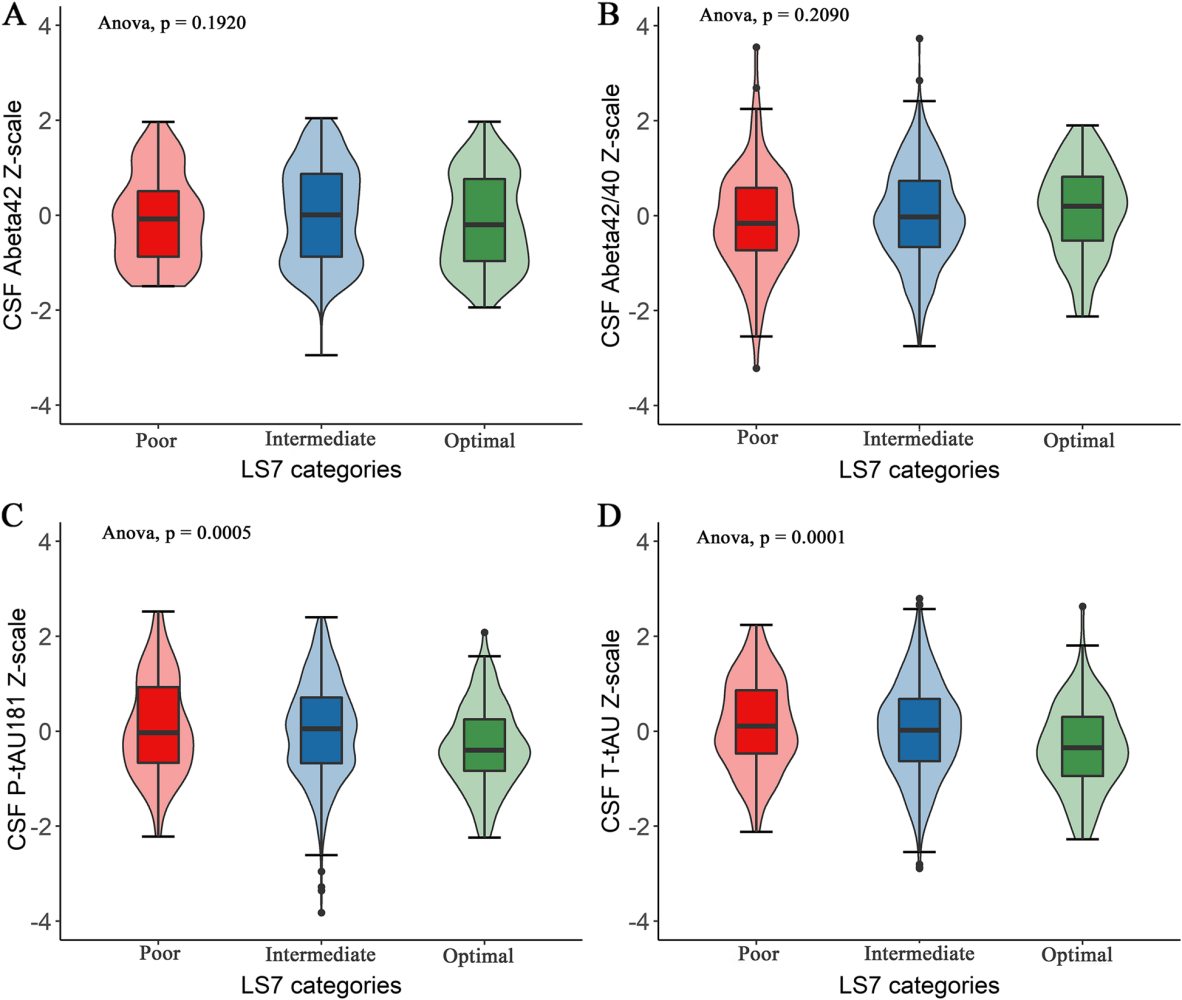
Differences in CSF biomarkers between the three LS7 categories. Differences in CSF Aβ42, Aβ42/40, p-tau181, and t-tau levels were examined by the analysis of variance. LS7, Life’s Simple 7; CSF, cerebrospinal fluid; Aβ, amyloid beta, P-tau181, phosphorylated tau181; T-tau, total tau

**Fig. 2:**
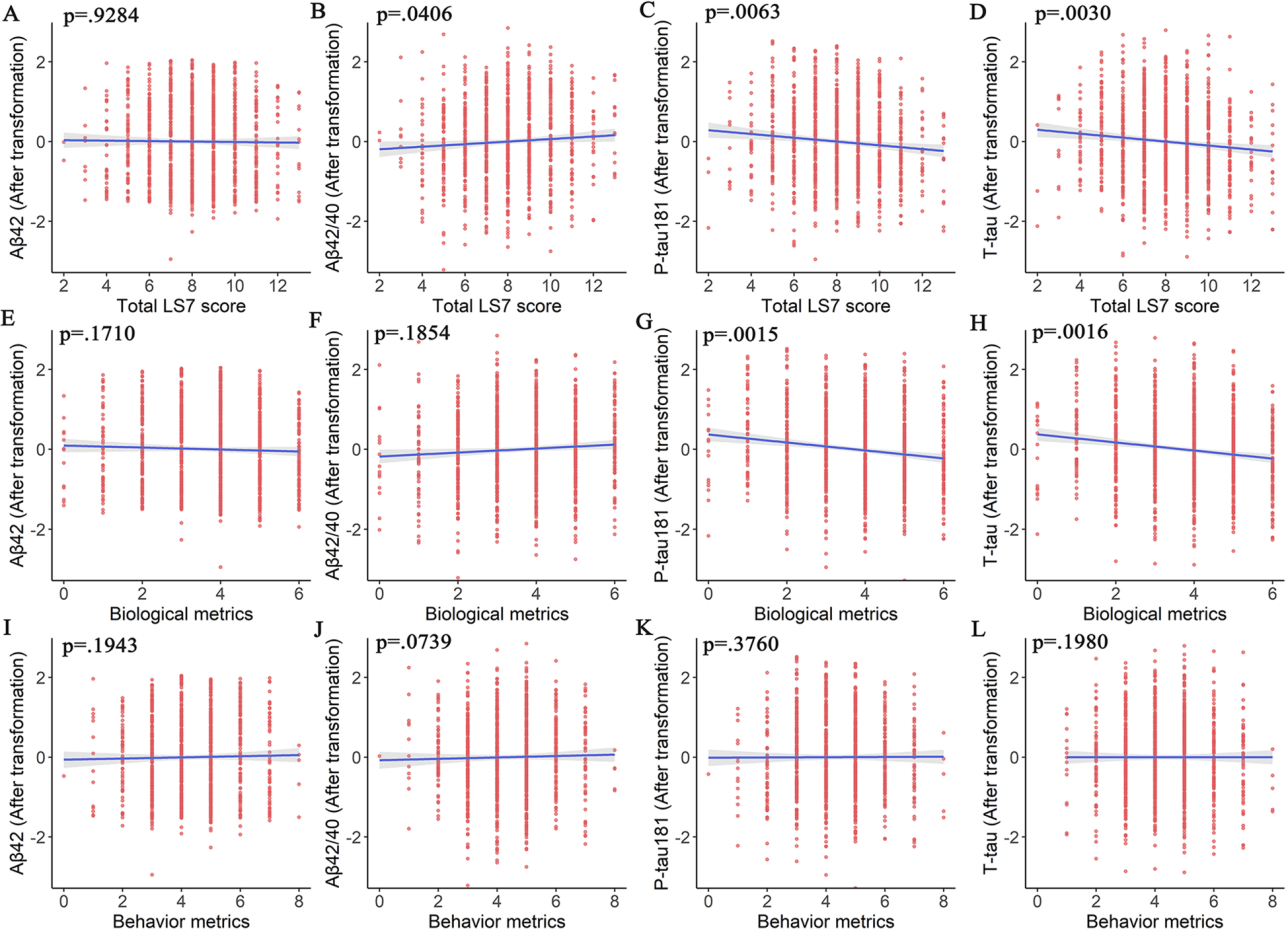
Associations between LS7 scores and CSF Aβ − and tau-related biomarkers. Multiple linear regression models were used to examine the associations between the total LS7 scores (**A**–**D**), subscales of biological metrics (**E**–**H**), and behavior metrics (**I**–**L**) with cerebrospinal fluid (CSF) Aβ42, Aβ42/40, p-tau181, and t-tau biomarkers, adjusting for age, sex, years of education, and *APOE* ɛ4 allele statuses. LS7, Life’s Simple 7; CSF, cerebrospinal fluid; Aβ, amyloid beta; P-tau181, phosphorylated tau181; T-tau, total tau

Across the three LS7 categories, the group with an optimal level of LS7 scores was proved to have lower CSF t-tau and p-tau181 levels (Fig. 1). Associations between higher LS7 scores and decreased CSF p-tau181 (*β* =  − 0.043, *P* = 0.006), t-tau (*β* =  − 0.044, *P* = 0.003), and increased Aβ42/40 (*β* = 0.034, *P* = 0.041) biomarkers were significantly revealed when adjusting for age, sex, education, and *APOE* ɛ4 allele (Fig. 2). In subscales, the biological metrics was significantly associated with p-tau181 (*β* =  − 0.073, *P* = 0.002) and t-tau (*β* =  − 0.069, *P* = 0.002) (Fig. 2). Besides, the relationships between the total LS7 score and the biological metrics with Aβ40 were also found (Additional file 1: Table S2). Yet, we did not record any association of behavior metrics. There were no significant associations with CSF Aβ42 (Fig. 2). Individually, we observed that the metrics of BP, glucose, and physical activity were related to CSF AD biomarkers (Additional file 1: Table S3).

### Interactions and stratified analyses by APOE ε4 allele statuses, age, and genders

**Fig. 3:**
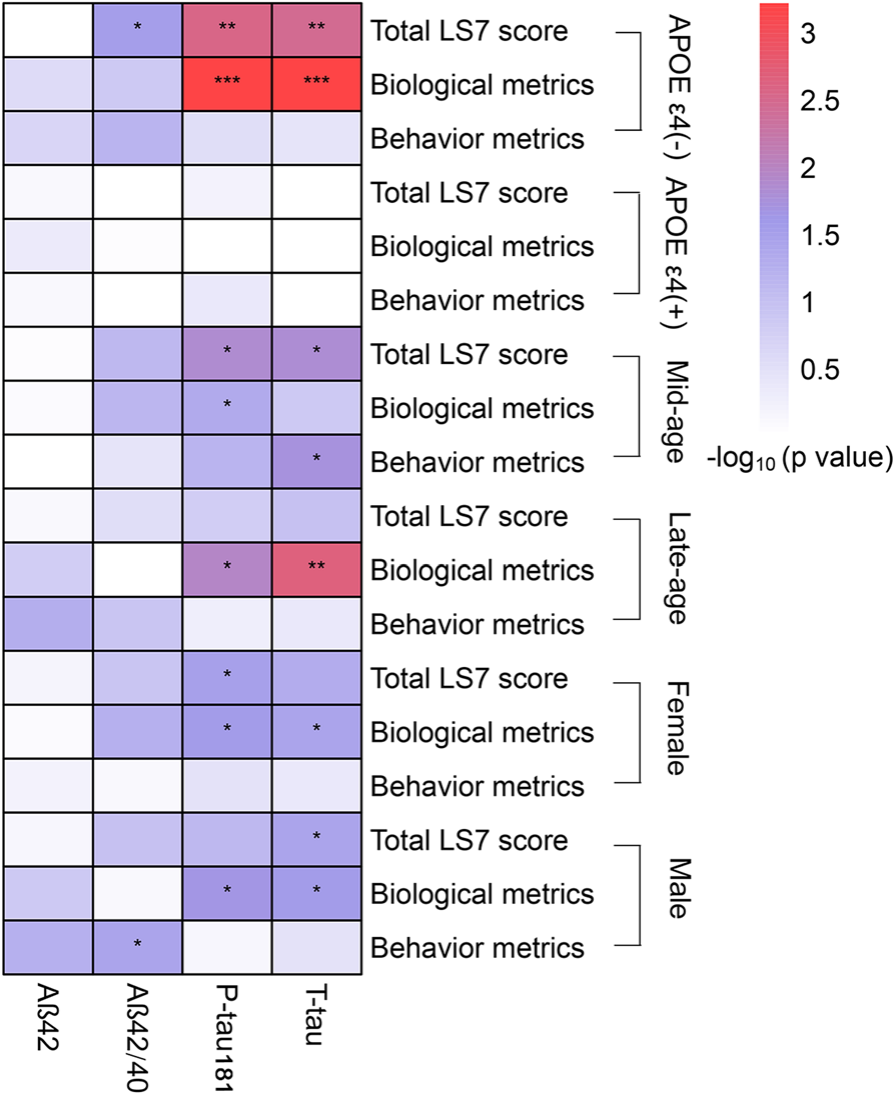
Subgroup analyses of associations between LS7 scores and CSF Aβ − and tau-related biomarkers stratified by *APOE* ɛ4 statuses, age, and sex. Multiple linear regression models were used to explore the associations with adjustment of age, sex, years of education, and *APOE* ɛ4 allele statuses. Asterisks represent statistical significance (*P < 0.05; **P < 0.01; ***P < 0.001). LS7, Life’s Simple 7; Aβ, amyloid beta; P-tau181, phosphorylated tau181; T-tau, total tau

Interaction between age and behavior metrics was found (*P* = 0.0243, Additional file 1: Table S4). In the mid-age, both biological and behavior metrics were significantly related to CSF tau-related biomarkers, whereas in the late age, only the biological metrics were noticed to be associated with CSF biomarkers (Fig. 3, Additional file 1: Table S5). Besides, significant associations between LS7 scores and CSF Aβ42/40 (*β* = 0.039, *P* = 0.029), p-tau181 (*β* =  − 0.049, *P* = 0.003), and t-tau (*β* =  − 0.050, *P* = 0.004) biomarkers were revealed among the *APOE* ε4 non-carriers, yet not among carriers (Fig. 3). Moreover, LS7 scores were associated with CSF AD biomarkers in both males and females, and the behavior metrics were observed to be associated with CSF Aβ42/40 in males (Fig. 3).

### Sensitivity analyses

We performed sensitivity analyses by additionally adjusting for the comorbidities of coronary heart disease and stroke and produced similar results of the associations between LS7 scores and CSF Aβ42/40, p-tau181, and t-tau biomarkers (Additional file 1: Table S6). Additionally, in the relatively healthy population with no history of hypertension, diabetes, and hyperlipemia, there was only a significant association between the biological metrics and CSF Aβ40 biomarker (Additional file 1: Table S7).

## Discussion

This study is the first to examine the association between LS7 scores and CSF AD biomarkers in the cognitively intact population. Our results demonstrated that LS7 scores, especially the biological metrics, were significantly related to CSF Aβ42/40 and tau-related pathology. These relationships were significant among the *APOE* ɛ4 non-carriers, yet not in the carriers. These findings provided supports for the linkage between LS7 cardiovascular health and AD risks.

Given the complicated nature of dementia, the interventions of LS7, which could control for multiple risk factors and underlying mechanisms at the same time, have been noticed to promote brain health and prevent dementia [4]. A 2-year randomized controlled trial demonstrated that multidomain intervention of vascular risks was beneficial to cognitive functioning for the at-risk elderly people [24]. In France, a cohort study by Samieri et al. indicated that optimal LS7 could reduce the incidence of dementia [25]. Other longitudinal studies from England, America, and Finland also suggested the associations between LS7 and cardiovascular health with dementia [10–12, 26]. Moreover, the relationship between a composite healthy lifestyle and risks of AD was observed in 2 longitudinal studies in Chicago [5]. Besides, the ideal cardiovascular health was found relevant to better cognitive performance [8, 9, 27], and less decline [7, 8, 25]. Nevertheless, not all results were consistent. In the Netherlands, a 6-year multidomain intervention of vascular care did not bring older people to a reduction of dementia. In that study, the population was not selected aged 70 years or older with modest cardiovascular risks at the baseline, which might be the reason for insignificance [16]. Also, no significant association between LS7 and dementia was found in Germany, which lacked the diet metric of their LS7 assessments [15]. Notably, there was still no examination of the relationship between LS7 and the pathology of AD.

In our CABLE study, we firstly revealed the relationship between LS7 scores and CSF Aβ42/40 even after additionally adjusting for the complications of coronary heart disease and stroke. That supported the correlations between LS7 score and AD risks despite the no significance with Aβ42, since many lines of evidence have suggested that CSF Aβ42/40 ratio performed better for discrimination of AD, which balanced the total production of amyloid beta peptides [28]. Besides, the decreased p-tau181 and t-tau indicated the relationship between LS7 scores and incipient AD risks. As CSF p-tau181 reflected the phosphorylation state of tau in brain, it is considered specific to AD pathologies. Total tau in CSF presented the neuronal damage and degeneration with less specificity for AD [29]. In our study, we have excluded the population with acute neurological disorders and other neurodegenerative diseases to reduce the potential heterogeneity. In sum, our findings provided pathological support for the association between LS7 and potential AD risks. Here, we involved all the 7 metrics of LS7, while the diet and physical activity metrics were limited to frequency. The population in the current study was relatively younger with intact cognitive function, which could represent the preclinical stage of AD. The correlations between LS7 and dementia and AD risks were verified, and the underlying mechanisms of accumulation of neurodegenerative pathology and the reduction of clearance may be involved in multiple pathways, including vascular risks, inflammatory, oxidative stress, and mitochondrial dysfunction [11, 30–33].

A few studies gazed into the summary and separate components of LS7, which could provide additional insights into the relationships. Both behavior and biological metrics of midlife were related to dementia incidence in London [10]. Based on a longitudinal cohort study in the USA, metabolic changes had a significant influence on late-life cognition [34]. Similarly, the biological metrics were suggested to be associated with cognitive decline and dementia in other two cohort studies, rather than the behavior metrics [7, 35]. In our study, the subscale of biologics was significantly associated with CSF Aβ − and tau-related biomarkers. Yet, we did not find the relationship of behavior metrics in the total population. The biological metric could reflect the metabolism of the human body objectively, which can be involved in inflammatory and immune mechanisms, and interact with genetic risks to activate neuropathology. Subjectivity in the measurements of behaviors might cause bias, and the effects of behavior metrics on cognition may involve other mechanisms such as psychological factors and cognitive reservation [36, 37]. Besides, the interaction between age and behavior metrics was found. In the mid-age, we found that both biological metrics and behavior metrics were relevant to CSF biomarkers, while in the late age, there was only biological metrics revealed significant association, which might be due to the complex effects of BMI on dementia attenuating the significance of behavior metrics in late life [38, 39]. Also, the association of biological metrics was only found in males, which could be derived from the different distribution of LS7 scores between the genders (Additional file 1: Fig. S1).

The genetic risks have been known as important factors contributing to the pathogenesis of dementia [40]. However, the interaction between lifestyle and genetic risk remains unclear [41]. From a series of longitudinal studies, associations between a composed lifestyle and dementia, AD, and cognition decline were suggested regardless of the *APOE* ε4 allele [5, 25, 42]. A 2-year multidomain intervention was found beneficial for both *APOE* ε4 non-carriers and carriers (Solomon et al. 2018). However, research in Rotterdam displayed the protective effects on dementia among low and intermediate genetic risk populations [41]. Also, significant associations between LS7 scores and composite lifestyles with dementia were observed only among *APOE* ε4 allele non-carriers, yet not in the carriers [11, 14]. The relationship between metabolic risk profile and cognitive performance was suggested stronger among *APOE* ε4 allele non-carriers [34]. In our CABLE study, we found the associations between LS7 scores, as well as the biological metrics and CSF AD biomarkers only among the *APOE* ɛ4 non-carriers, yet we failed to record these associations in the carriers. The population in our study was relatively younger (mean age = 62.71 years), and the influences of the *APOE* ε4 allele on dementia were suggested different, attenuating with increasing age, which might explain part of the non-significant interactions [43]. Nonetheless, the number of *APOE* ε4 carriers in our study was limited and only 137 (13.92%), which might lead to a false negative. More researches with an ample sample size and a sufficiently long follow-up period are necessary.

### Limitations

Some limitations should be noted. This study was cross-sectional. The information on diet and physical activity metrics were based on frequency lacking quantitative data with only two dietary ingredients of fruit and fish, which were major weaknesses comparing with the standard AHA criteria. Besides, there was only a significant association with Aβ40 in the sensitivity analyses of the relatively healthy population without histories of hypertension, diabetes, and hyperlipemia. Moreover, in these populations, the opposite relationship with Aβ42 existed, which may be due to the bias by poor efficacy of LS7 for discrimination, and the longitudinal studies with the large sample are needed for further exploration. All analyses were performed on people recruited from the hospital, which can be further researched with a community-based population in the future.

## Conclusions

In summary, LS7 scores were significantly associated with CSF Aβ42/40, p-tau181, and t-tau biomarkers of AD in the cognitively intact population, which offered a pathological verification of multidomain intervention. Therefore, putting efforts into improving LS7 cardiovascular health might be helpful to prevent AD, especially in the *APOE* ɛ4 non-carriers. More longitudinal researches with a larger sample size as well as randomized controlled trials are anticipated in the future.

## Supplementary Information

**Additional file 1: Table S1.** Modified measurements of Life’s Simple 7 in our study. **Table S2.** Associations between LS7 scores with CSF AD biomarkers. **Table S3.** Associations between individual component of LS7 with CSF AD biomarkers. **Table S4.** Interaction analyses by APOE ɛ4 genotype, age, and genders. **Table S5.** Subgroup analyses of associations between total LS7 and subscales with CSF AD biomarkers. **Table S6.** Sensitivity analyses of associations between LS7 scores with CSF AD biomarkers additionally adjusting for comorbidities. **Table S7.** Sensitivity analyses of associations between LS7 scores with CSF AD biomarkers in the population with no history of hypertension, diabetes, and hyperlipemia. **Fig. S1.** The distribution of each component scores of LS7.

## Data Availability

The datasets used and analyzed in the current study are available from the corresponding authors on reasonable request.

## Abbreviations

LS7: Life’s Simple 7
CSF: Cerebrospinal fluid
AD: Alzheimer’s disease
CABLE: Chinese Alzheimer’s Biomarker and LifestylE study
*APOE* ε4: Apolipoprotein E genotype ε4
Aβ: Amyloid β plaques
p-tau181: Phosphorylated tau181
t-tau: Total tau
AHA: American Heart Association
CV: Coefficient of variation
SD: Standard deviation
CM-MMSE: China Modified Mini-Mental State Examination
HAMD: Hamilton Rating Scale for Depression
HAMA: Hamilton Rating Scale for Anxiety
BMI: Body mass index
BP: Blood pressure
TC: Total cholesterol
