# Supplementary material for: Association between Life’s Simple 7 and cerebrospinal fluid biomarkers of Alzheimer’s disease pathology in cognitively intact adults: the CABLE study
Source: Alzheimers Res Ther. 2022 May 26;14:74. doi: 10.1186/s13195-022-01019-2 (PMC9134665; doi:10.1186/s13195-022-01019-2)
Supplement: Supplementary file 1 — Additional file 1: Table S1. Modified measurements of Life’s Simple 7 in our study. Table S2. Associations between LS7 scores with CSF AD biomarkers. Table S3. Associations between individual component of LS7 with CSF AD biomarkers. Table S4. Interaction analyses by APOE ɛ4 genotype, age, and genders. Table S5. Subgroup analyses of associations between total LS7 and subscales with CSF AD biomarkers. Table S6. Sensitivity analyses of associations between LS7 scores with CSF AD biomarkers additionally adjusting for comorbidities. Table S7. Sensitivity analyses of associations between LS7 scores with CSF AD biomarkers in the population with no history of hypertension, diabetes, and hyperlipemia. Fig. S1. The distribution of each component scores of LS7. [file 13195_2022_1019_MOESM1_ESM.docx]

**Supplementary Materials**

**Supplementary Table 1.** Modified measurements of Life’s Simple 7 in our study

**Supplementary Table 2.** Associations between LS7 scores with CSF AD biomarkers

**Supplementary Table 3.** Associations between individual component of LS7 with CSF AD biomarkers

**Supplementary Table 4.** Interaction analyses by APOE ɛ4 genotype, age, and genders

**Supplementary Table 5.** Subgroup analyses of associations between total LS7 and subscales with CSF AD biomarkers

**Supplementary Table 6.** Sensitivity analyses of associations between LS7 scores with CSF AD biomarkers additionally adjusting for comorbidities

**Supplementary Table 7.** Sensitivity analyses of associations between LS7 scores with CSF AD biomarkers in the population with no history of hypertension, diabetes, and hyperlipemia

**Supplementary Fig. 1.** The distribution of each component scores of LS7

**Supplementary Table 1.** Modified measurements of Life’s Simple 7 in our study

| Goal/Metric | Poor Health (score = 0) | Intermediate Health (score = 1) | Ideal Health (score = 2) |
| --- | --- | --- | --- |
| Blood pressure  (The mean of first 5 days of hospitalizations) | SBP ≥ 140 or DBP ≥ 90 mmHg | SBP 120-139 or DBP 80-89 mmHg or  SBP < 120 and DBP < 80 mmHg treated | SBP < 120 and DBP < 80 mmHg untreated |
| Total cholesterol | ≥ 240 mg/dL | 200-239 mg/dL or < 200 mg/dL treated | < 200 mg/dL untreated |
| Blood glucose | ≥ 126 mg/dL | Glucose: 100-125 mg/dL or < 100 mg/dL treated | < 100 mg/dL untreated |
| Body mass index (BMI) | ≥30 kg/m^2^ | 25-30 kg/m^2^ | < 25.0 kg/m^2^ |
| Smoke | Current smoking or quit smoking for less than five years | Quit smoking for more than five years | Never smoking |
| Diet  Based on the eating frequency for the two components  1) Fruit  2) Fish   - never or occasionally coded as 0 for each metric - once or several times a week coded as 1 for each metric - every day coded as 2 for each metric | Total score of 0 or 1 of the two components (0-1/4) | Total score of 2 or 3 of the two components (2-3/4) | Total score of 4 of two components (4/4) |
| Physical activity  Based on the frequency in leisure-time | Never or occasionally | Once a week or several times a week | Every day |

DBP, diastolic blood pressure; SBP, systolic blood pressure;

**Supplementary Table 2.** Associations between LS7 scores with CSF AD biomarkers

| Variable | Aβ42 | | Aβ40 | | Aβ42/40 | | P-tau181 | | T-tau | |
| --- | --- | --- | --- | --- | --- | --- | --- | --- | --- | --- |
|  | β | p | β | p | β | p | β | p | β | p |
| Total LS7 scores | -0.0015 | .9284 | -0.0465 | **.0039** | 0.0343 | **.0406** | -0.0431 | **.0063** | -0.0442 | **.0030** |
| Subscale |  |  |  |  |  |  |  |  |  |  |
| Biological metrics | -0.0329 | .1710 | -0.0792 | **.0008** | 0.0325 | .1854 | -0.0731 | **.0015** | -0.0691 | **.0016** |
| Behavior metrics | 0.0328 | .1943 | -0.0239 | .3449 | 0.0468 | .0739 | -0.0219 | .3760 | -0.0302 | .1980 |

Multiple linear regression models were conducted with all models adjusted for age, sex, education, and *APOE* ε4 status.

Aβ, amyloid β; P-tau181, phosphorylated tau181; T-tau, total tau protein

The statistically significant results were bolded.

**Supplementary Table 3.** Associations between individual component of LS7 with CSF AD biomarkers

| Variable | Aβ42 | | Aβ40 | | Aβ42/40 | | P-tau181 | | T-tau | |
| --- | --- | --- | --- | --- | --- | --- | --- | --- | --- | --- |
|  | β | p | β | p | β | p | β | p | β | p |
| BP | -0.0431 | .3304 | -0.1921 | **<.0001** | 0.1290 | **.0046** | -0.1493 | **.0005** | -0.1576 | **.0001** |
| Total cholesterol | -0.0583 | .2295 | 0.0031 | .9490 | -0.0342 | .4944 | 0.0288 | .5419 | 0.0552 | .2170 |
| Glucose | -0.0193 | .6587 | -0.0822 | .0555 | 0.0110 | .8050 | -0.1247 | **.0032** | -0.1246 | **.0019** |
| BMI | -0.0764 | .1352 | -0.0676 | .1850 | 0.0004 | .9933 | -0.0005 | .9912 | -0.0097 | .8383 |
| Smoking | 0.0602 | .1794 | -0.0247 | .5852 | 0.0540 | .2459 | -0.0184 | .6743 | -0.0185 | .6557 |
| Diet | -0.1804 | .0654 | 0.1497 | .1285 | -0.2534 | **.0121** | -0.0555 | .5660 | -0.0717 | .4316 |
| Physical activity | -0.1804 | .0654 | -0.0222 | .5690 | 0.1096 | **.0063** | -0.0288 | .4480 | -0.0409 | .2600 |

Multiple linear regression models were conducted with all models adjusted for age, sex, education, and *APOE* ε4 status.

Aβ, amyloid β; T-tau, total tau protein; P-tau181, phosphorylated tau181; BP, blood pressure; Glucose, fasting blood glucose; BMI, body mass index

The statistically significant results were bolded.

**Supplementary Table 4.** Interaction analyses by *APOE* ɛ4 genotype, age, and genders.

|  | Aβ42 | | Aβ40 | | Aβ42/40 | | P-tau181 | | T-tau | |
| --- | --- | --- | --- | --- | --- | --- | --- | --- | --- | --- |
| Subgroups | β | p | β | p | β | p | β | p | β | p |
| *APOE*ɛ4 genotype x |  |  |  |  |  |  |  |  |  |  |
| Total LS7 score | -0.0080 | .8580 | 0.0191 | .6566 | -0.0257 | .5716 | 0.0424 | .3204 | 0.0253 | .5342 |
| Biological metrics | 0.0089 | .8930 | 0.0393 | .5382 | -0.0071 | .9144 | 0.0619 | .3259 | 0.0690 | .2504 |
| Behavior metrics | -0.0277 | .6955 | -0.0041 | .9527 | -0.0517 | .4748 | 0.0299 | .6601 | -0.0209 | .7480 |
| Age x |  |  |  |  |  |  |  |  |  |  |
| Total LS7 score | 0.0021 | .1927 | 0.0016 | .2847 | 0.0003 | .8183 | 0.0001 | .9400 | 0.0001 | .9149 |
| Biological metrics | -0.0010 | .6828 | 0.0020 | .3713 | -0.0036 | .1437 | 0.0003 | .8756 | -0.0025 | .2439 |
| Behavior metrics | 0.0051 | **.0243** | 0.0020 | .3813 | 0.0037 | .1186 | 0.0005 | .8355 | 0.0032 | .1295 |
| Sex x |  |  |  |  |  |  |  |  |  |  |
| Total LS7 scores | 0.0295 | .3732 | 0.0110 | .7339 | -0.0007 | .9828 | 0.0201 | .5308 | 0.0128 | .6705 |
| Biological metrics | -0.0251 | .5930 | 0.0158 | .7305 | -0.0501 | .2958 | 0.0032 | .9434 | 0.0135 | .7516 |
| Behavior metrics | 0.0865 | .1172 | -0.0024 | .9638 | 0.0532 | .3486 | 0.0376 | .4879 | 0.0108 | .8340 |

Interactions were examined by the terms of *APOE*ɛ4 genotype, age and genders with LS7 in fully adjusted regression models.

Aβ, amyloid β; T-tau, total tau protein; P-tau181, phosphorylated tau181

The statistically significant results were bolded.

**Supplementary Table 5.** Subgroup analyses of associations between total LS7 and subscales with CSF AD biomarkers

|  | Aβ42 | | Aβ40 | | Aβ42/40 | | P-tau181 | | T-tau | |
| --- | --- | --- | --- | --- | --- | --- | --- | --- | --- | --- |
| Subgroups | β | p | β | p | β | p | β | p | β | p |
| *APOE*ɛ4 non-carriers |  |  |  |  |  |  |  |  |  |  |
| Total LS7 score | 0.0010 | .9574 | -0.0490 | **.0052** | 0.0392 | **.0291** | -0.0496 | **.0027** | -0.0503 | **.0035** |
| Biological metrics | -0.0295 | .2620 | -0.0856 | **.0008** | 0.0386 | .1434 | -0.0832 | **.0006** | -0.0856 | **.0006** |
| Behavior metrics | 0.0343 | .2108 | -0.0218 | .4269 | 0.0514 | .0660 | -0.0272 | .2950 | -0.0247 | .3580 |
| *APOE*ɛ4 carriers |  |  |  |  |  |  |  |  |  |  |
| Total LS7 score | -0.0142 | .7440 | -0.0381 | .3519 | -0.0015 | .9702 | -0.0185 | .6280 | -0.0018 | .9618 |
| Biological metrics | -0.0478 | .4510 | -0.0480 | .4298 | -0.0069 | .9090 | 0.0007 | .9899 | -0.0022 | .9691 |
| Behavior metrics | 0.0211 | .7620 | -0.0433 | .5155 | 0.0045 | .9470 | -0.0496 | .4223 | -0.0022 | .9719 |
| Mid-age |  |  |  |  |  |  |  |  |  |  |
| Total LS7 scores | -0.0032 | .8768 | -0.0507 | **.0126** | 0.0378 | .0786 | -0.0496 | **.0136** | -0.0473 | **.0148** |
| Biological metrics | -0.0077 | .8053 | -0.0662 | **.0309** | 0.0583 | .0726 | -0.0603 | **.0466** | -0.0430 | .1406 |
| Behavior metrics | 0.0004 | .9912 | -0.0562 | .0875 | 0.0314 | .3619 | -0.0594 | .0664 | -0.0733 | **.0190** |
| Late-age |  |  |  |  |  |  |  |  |  |  |
| Total LS7 scores | 0.0086 | .7517 | -0.0325 | .2317 | 0.0292 | .2939 | -0.0375 | .1658 | -0.0418 | .1057 |
| Biological metrics | -0.0533 | .1634 | -0.0837 | **.0292** | 0.0004 | .9918 | -0.0956 | **.0115** | -0.1120 | **.0022** |
| Behavior metrics | 0.0779 | .0526 | 0.0207 | .6100 | 0.0647 | .1188 | 0.0248 | .5384 | 0.0306 | .4330 |
| Female |  |  |  |  |  |  |  |  |  |  |
| Total LS7 scores | -0.0122 | .6472 | -0.0483 | .0576 | 0.0421 | .1180 | -0.0582 | **.0311** | -0.0482 | .0503 |
| Biological metrics | -0.0082 | .8200 | -0.0835 | **.0172** | 0.0701 | .0571 | -0.0809 | **.0273** | -0.0706 | **.0369** |
| Behavior metrics | -0.0248 | .6025 | -0.0151 | .7467 | 0.0158 | .7480 | -0.0461 | .3414 | -0.0358 | .4270 |
| Male |  |  |  |  |  |  |  |  |  |  |
| Total LS7 scores | 0.0079 | .7066 | -0.0435 | **.0406** | 0.0359 | .1017 | -0.0348 | .0788 | -0.0398 | **.0366** |
| Biological metrics | -0.0489 | .1372 | -0.0723 | **.0264** | 0.0091 | .7884 | -0.0694 | **.0223** | -0.0648 | **.0269** |
| Behavior metrics | 0.0571 | .0575 | -0.0264 | .3873 | 0.0656 | **.0362** | -0.0114 | .6930 | -0.0262 | .3440 |

Multiple linear regression models were conducted with all models adjusted for age, sex, education, and *APOE* ε4 status.

Aβ, amyloid β; T-tau, total tau protein; P-tau181, phosphorylated tau181;

The statistically significant results were bolded.

**Supplementary Table 6.** Sensitivity analyses of associations between LS7 scores with CSF AD biomarkers additionally adjusting for comorbidities

| Variable | Aβ42 | | Aβ40 | | Aβ42/40 | | P-tau181 | | T-tau | |
| --- | --- | --- | --- | --- | --- | --- | --- | --- | --- | --- |
|  | β | p | β | p | β | p | β | p | β | p |
| Total LS7 scores | 0.0039 | .8098 | -0.0455 | **.0052** | 0.0380 | **.0245** | -0.0397 | **.0131** | -0.0444 | **.0033** |
| Subscales |  |  |  |  |  |  |  |  |  |  |
| Biological metrics | -0.0265 | .2744 | -0.0777 | **.0012** | 0.0369 | .1372 | -0.0681 | **.0036** | -0.0696 | **.0017** |
| Behavior metrics | 0.0381 | .1314 | -0.0230 | .3663 | 0.0503 | .0554 | -0.0188 | .4481 | -0.0294 | .2130 |

Sensitivity analyses were conducted when additionally adjusting for history of coronary heart disease and stroke.

Aβ, amyloid β; T-tau, total tau protein; P-tau181, phosphorylated tau181

The statistically significant results were bolded.

**Supplementary Table 7.** Sensitivity analyses of associations between LS7 scores with CSF AD biomarkers in the population with no history of hypertension, diabetes, and hyperlipemia

| Variable | Aβ42 | | Aβ40 | | Aβ42/40 | | P-tau181 | | T-tau | |
| --- | --- | --- | --- | --- | --- | --- | --- | --- | --- | --- |
|  | β | p | β | p | β | p | β | p | β | p |
| Total LS7 scores | -0.0147 | .5320 | -0.0355 | .1294 | 0.0164 | .5052 | -0.0291 | .1984 | -0.0354 | .1023 |
| Subscales |  |  |  |  |  |  |  |  |  |  |
| Biological metrics | -0.1134 | **.0032** | -0.0786 | **.0399** | -0.0128 | .7529 | -0.0365 | .3245 | -0.0521 | .1404 |
| Behavior metrics | 0.0545 | .1007 | -0.0122 | .7112 | 0.0423 | .2256 | -0.0306 | .3374 | -0.0315 | .3029 |

Analyses were adjusted for age, sex, education, and *APOE* ε4 status.

Aβ, amyloid β; T-tau, total tau protein; P-tau181, phosphorylated tau181

The statistically significant results were bolded.

**Supplementary Fig. 1.** The distribution of each component scores of LS7


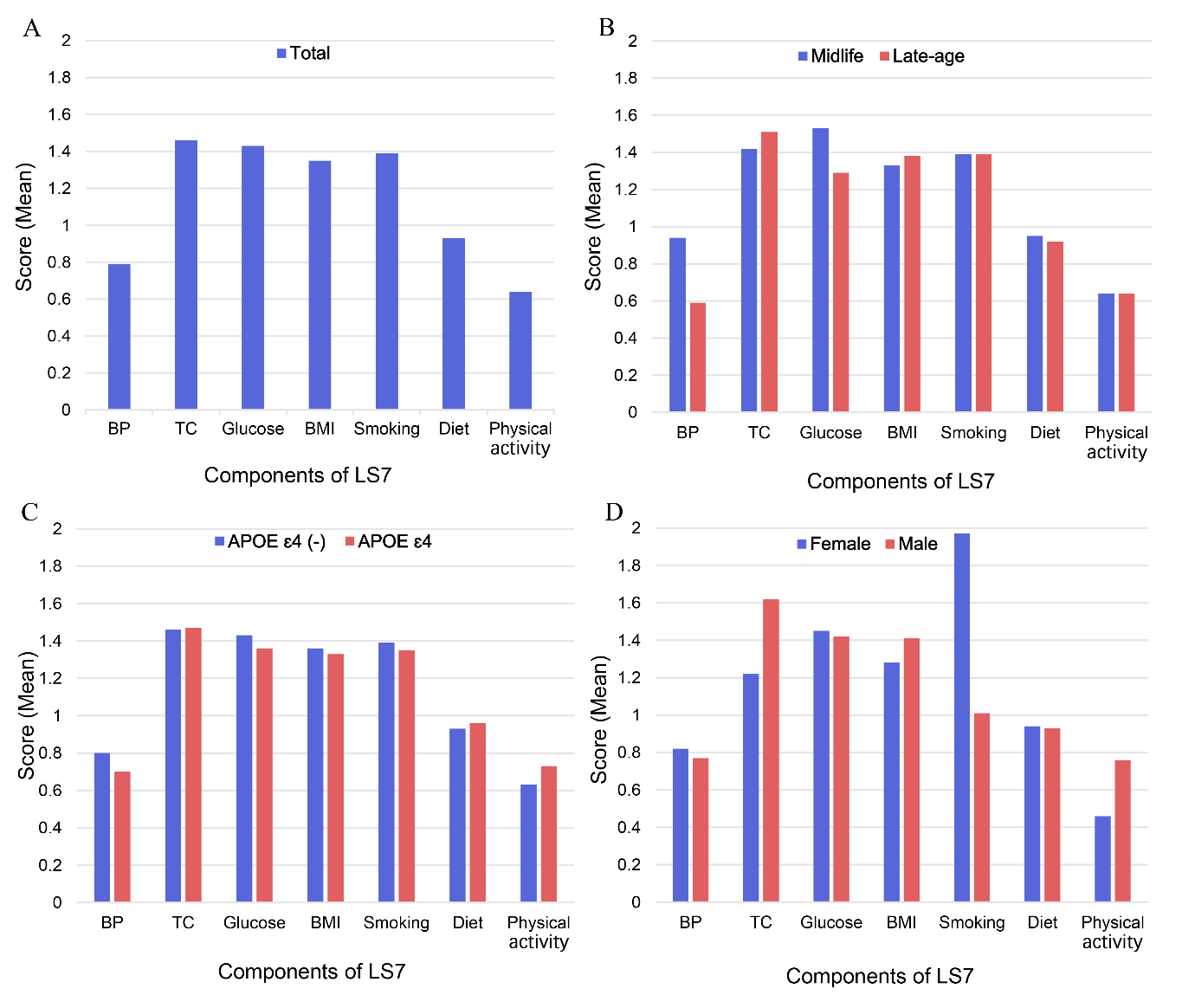


The distribution of each component scores of LS7 in total population (A), in midlife and late-age population (B), in *APOE* ε4 non-carriers and *APOE* ε4 carriers (C), and in female and male (D).

LS7, Life’s Simple 7; BP, blood pressure; TC, total cholesterol; Glucose, fasting blood glucose; BMI, body mass index
